# Feasibility and reliability of measuring muscle stiffness in Parkinson's Disease using MyotonPRO device in a clinical setting in Ghana

**DOI:** 10.4314/gmj.v56i2.4

**Published:** 2022-06

**Authors:** Mary W Agoriwo, Paul E Muckelt, Cynthia O Yeboah, Beatrice E A Sankah, Sandra Agyapong-Badu, Albert Akpalu, Maria Stokes

**Affiliations:** 1 Physiotherapy Department, Korle Bu Teaching Hospital, Accra, Ghana; 2 School of Health Sciences, University of Southampton, UK; 3 Centre for Sport, Exercise and Osteoarthritis Research Versus Arthritis, University of Southampton, UK; 4 School of Sport, Exercise and Rehabilitation Sciences, University of Birmingham, U.K; 5 Department of Medicine, University of Ghana Medical School/KBTH; 6 Department of Physiotherapy and Rehabilitation Sciences, University of Health and Allied Sciences, Ho, Ghana; 7 Southampton NIHR Biomedical Research Centre, Southampton General Hospital, Southampton, UK

**Keywords:** Parkinson's disease, Muscle stiffness, Assessment, Feasibility, Reliability

## Abstract

**Objectives:**

To examine the feasibility and within-session reliability of Myoton technology to measure muscle stiffness objectively in people with Parkinson's disease in an outpatient setting

**Design:**

An exploratory quantitative study design was used.

**Setting:**

The study was conducted in the outpatient physiotherapy department of a Teaching Hospital in Ghana. Participants were recruited from three sites.

**Participants:**

Thirty adults with Parkinson's disease over 18 years with increased tone (muscle stiffness) and at Hoehn and Yahr Stages I-III were studied. Persons with severe comorbidities were excluded.

**Intervention:**

There was no intervention before testing. The MyotonPRO device measured the mechanical properties of the biceps brachii, flexor carpi radialis and tibialis anterior muscles in a relaxed supine position. The probe applied mechanical impulses to the skin, eliciting tissue oscillations. The muscles' three parameters (stiffness, non-neural tone and elasticity) were recorded bilaterally. The reliability of two sets (of 5 impulses) of Myoton data on all three muscles was examined.

**Results:**

All 30 participants (66.3±8.9 years) were recruited and tested within eight weeks. Intraclass correlation coefficients (ICC 3,2) were above 0.92 for biceps brachii and tibialis anterior and above 0.86 for flexor carpi radialis.

**Conclusion:**

The MyotonPRO was reliable for measuring two sets of data within the same session, indicating that only one set of measurements is needed. The technique is feasible and easy to use in a clinical setting in Ghana, with the potential to assess the effect of medical and physiotherapy interventions on muscles in people with Parkinson's disease.

**Funding:**

M.S discloses a grant from the Science and Technology Facilities Council Impact Acceleration Account at the University of Southampton to support this collaborative research (no personal finance received). All other authors, M.W.A, P.E.M, C.O.Y, B.E.A.S, S.A.-B, and A.A have no financial disclosure

## Introduction

Parkinson's disease (PD) is a progressive neurodegenerative disorder characterised by four cardinal motor signs including bradykinesia, rest tremors, rigidity and postural instability due to loss of dopaminergic cells in the substantia nigra.^1^ Globally, PD is the second most common neurodegenerative disorder following Alzheimer's disease,^2^ with numerous challenging symptoms. PD is associated with a prodrome of non-motor symptoms such as loss of smell and taste, fatigue, inattention, excessive sweating and constipation, which persist with disease progression.^3^ PD usually affects the older population of 60 years and above, with a 1.3% prevalence rate in industrialised countries.^4^

However, it also has a young onset of 40 and a juvenile onset of 20.^4^ A 60%–80% depletion in striatal dopamine results in apparent motor symptoms of PD.^5^

The symptoms of PD degenerate into variable physical impairments with poor balance, reduced physical activity and severe walking impairments,^6^ which eventually lead to an increase in falls, loss of independence and immobility.^7^ Medication and physical treatments used to reduce tone in PD are difficult to assess clinically.^8^,^9^ Increased muscle tone (including rigidity) is experienced by the patient as stiffness and sometimes muscular pain and can severely restrict a person's ability to carry out functional activities.^10^ Muscle tone is assessed subjectively, usually tested manually by passive flexion and extension of the wrist or elbow, and rated as mild, moderate or severe.^11^ Therefore, a robust method that is objective, accurate and reliable for assessing changes in muscle tone or stiffness is needed. The present study addressed this challenge using a novel technology termed Myoton.

Myoton technology offers an objective, non-invasive alternative to subjective assessment by measuring the mechanical properties of muscle using a hand-held device that is relatively easy to use. The MyotonPRO device has a rounded probe that applies a gentle mechanical impulse to the skin over a muscle, which induces oscillations of the muscle recorded by the device. Various parameters are calculated simultaneously, including non-neural tone, stiffness and elasticity. A potential clinical benefit of Myoton technology is its use for measuring changes in muscle tone in neurological disorders, particularly stroke^12^ and PD.^13^,^14^ Myoton technology has been studied in people with PD to measure increased stiffness of muscle and tendons.^13^,^15^,^16^

Important properties of an assessment tool are reliability and validity. The reliability of Myoton technology is reported to be generally good in studies of healthy,^17^,^18^ and clinical populations, such as stroke.^12^ The MyotonPRO device has been validated against electromyography (EMG) and force in healthy muscles.^18^ In persons with PD, criterion validity was established for Myoton against EMG^13^, and the parameters were valid compared to clinical rigidity scores.^14^ The robustness of the MyotonPRO device needs to be examined in challenging environments and has been demonstrated as such in astronauts in microgravity.^19^ Most Myoton studies have been con-ducted in laboratory environments, so feasibility in clinical environments needs to be examined.

Another important requirement of an assessment tool is the ability to measure the effect of interventions, i.e. sensitivity to change.

The Myoton device has demonstrated this ability in persons with PD by showing a reduction in muscle stiffness after brain stimulation^14^ and medication.^16^ However, Myoton technology is yet to be used among the African population in a clinical environment. Hence, this study sought to investigate the reliability and feasibility of the MyotonPRO device among people with PD in Ghana.

The aim was to examine the feasibility and reliability of using the MyotonPRO device to measure stiffness in persons with PD in an outpatient setting in Africa (Ghana). The objectives were to examine: 1) the feasibility of using the device in routine clinical practice in Ghana; 2) the reliability of Myoton parameter recordings within the same session for measuring muscle stiffness in people with PD, to see if two sets of recordings are needed.

## Methods

The study was an exploratory quantitative study of people with PD with increased tone or muscle stiffness to investigate the reliability of the MyotonPRO device in measuring changes in the mechanical properties of a muscle, such as tone. Patient and public involvement (PPI) activities were also undertaken through discussions with patients and physiotherapists to assess the feasibility and acceptability of the MyotonPRO technology.

### Participants and Recruitment

The study recruited 30 individuals with PD in Hoehn and Yahr Stages I-III using convenience sampling.

**a. Inclusion criteria** - males and females over 18 years, diagnosed as having PD and increased tone (muscle stiffness).

**b. Exclusion criteria** - history of severe limb or spinal injury, skin conditions; skeletal muscle relaxant in current use or medication that affects muscle tone or contractile ability; BMI.>30^20^ and those unable to understand study requirements.^21^

**c. Recruitment**

Before enrolling participants, ethical approval was obtained from the Korle Bu Teaching Hospital (KBTH) ethics review board (Ref No. KBTH-IRB/000121/2018). Recruitment was carried out at the Neurology Clinic and Physiotherapy Department of the Korle Bu Teaching Hospital, Accra, and from the PD Support Group Ghana. Posters advertising the study were placed in appropriate areas around the hospital, with permission to display posters sought as required from heads of departments. The poster was also posted on the common WhatsApp platform of PD Support Group Ghana members.

The details of a contact person running the study were provided on the posters to allow interested participants to contact the study team. Individuals had a minimum of 24 hours, and up to a week, to decide and indicate if they wished to participate in the study. They were then contacted to determine their suitability for the study and to arrange a convenient time for testing. A participant information sheet and consent form were provided before data collection, including the study's purpose and methods and information on how to withdraw. Data were collected for eight weeks.

### Equipment

**Figure 1:**
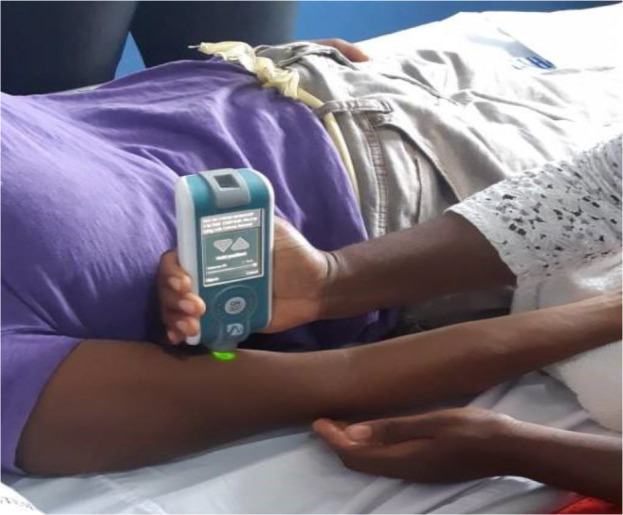
MyotonPRO device in use for assessing the mechanical properties of the Biceps Brachii muscle.

**Figure 2:**
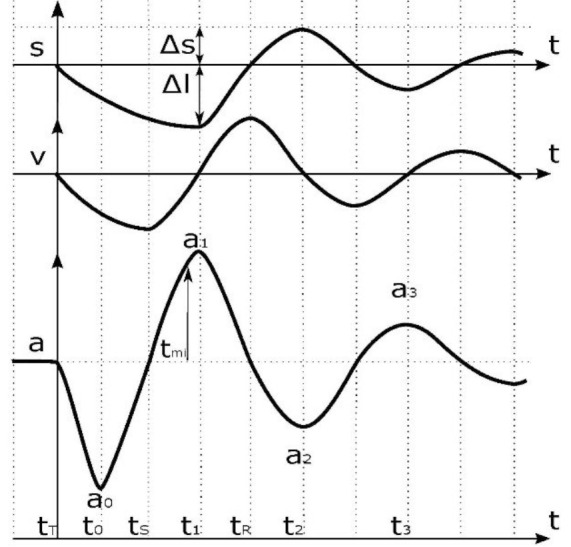
Waveforms demonstrating muscle oscillation and its relative displacement (S) and velocity (V) in relation to the oscillation acceleration (a).

The MyotonPRO (Myoton AS, Estonia) is a wireless hand-held device (Figure 1) that applies a brief (15 milliseconds) mechanical tap (impulse) to the skin over a muscle via a rounded probe, causing damped oscillations which are recorded by the device (Figure 2).^22^ The MyotonPRO device was approved for use in Ghana by the Ghana Standards Authority before data collection. The low force (0.4 Newtons) mechanical impulse is applied with constant preload (0.18 Newtons) independently of the investigator to pre-compress subcutaneous tissues. The three main parameters recorded from the oscillations are tone, stiffness and elasticity.^23^ Non-neural tone, or state of tension, is calculated automatically from signal spectrum Fmax (FFT) - (FFT — Fast Fourier Transform) and is recorded as frequency (Hz) of the damped oscillations. Stiffness is measured in Newton/meter (N/m) and represents the muscle's ability to resist an external force that modifies its shape.^24^ Elasticity is characterised by the logarithmic decrement (log) of the dampened oscillations, reflecting the ability of the tissue to recover its shape after being deformed.

a = Acceleration signal of the natural damped oscillation — unit: mG (1G = 1000mG); a_0_ = Maximum acceleration; a_1_ = Maximum displacement i.e. maximum tissue resistance measured in mG; a_2_ = Maximum opposite displacement due to inertia of the oscillation; a_3_ = Maximum displacement of the second period of the oscillation; ΔS = Pre-compression of subcutaneous tissues above a muscle being measured; Δl = Maximum displacement of a tissue being measured; S = Displacement (tissue oscillation, unit: mm); t = Time — unit: ms (1s = 1000ms); t_mi_ = The end of mechanical impulse; t_0_ = The time-point of maximum acceleration reached; t_T_ = Initiation of the mechanical impulse (measurement impulse); t_r_ = The time-point of tissue recovery to initial shape; t_s_ = Zero acceleration and maximum velocity; t_1_ = The time-point of maximum displacement; t_2_ = The time-point of maxi-mum opposite displacement; t_3_ = The time-point of maximum displacement of the second period of oscillation; V = Oscillation velocity-unit: m/s (Diagram provided courtesy of Myoton AS, Estonia).

### Myoton recording sites

Three muscles of the upper and lower limbs were tested:

- Upper arm flexors - biceps brachii
- Forearm flexors - flexor carpi radialis
- Foot dorsiflexors - tibialis anterior

The muscles were tested with the participant relaxed in supine lying. The recording sites were located following procedures in published studies as described below. 25,23,12 The three muscle sites were tested on both the right and left sides of the body.

### Biceps brachii

Participant positioning: relaxed supine, with the shoulder, externally rotated, elbow extended, and wrist supinated (hand facing upwards). A sandbag/small pillow is placed under the wrist to flex the elbow 15–20 degrees to take the stretch off the muscle and enable relaxation.

*Location of test site:* 75% of the distance between the lateral tip of the acromion and the mid cubital fossa. The middle of the muscle belly does not always fall along this longitudinal line, so is identified by palpation and visual observation during a low force contraction of biceps, as discussed by Agyapong et al.^25^

### Flexor carpi radialis

Participant positioning: relaxed supine, with the arm by the side, the forearm supinated (palm facing upwards), and the elbow flexed to approximately 40 degrees to take the stretch off the forearm flexors.

*Location of test site:* 33% (a third) of the distance between the elbow crease and lateral side of the ulnar styloid. The middle of the muscle belly is palpated during a gentle isometric contraction while the investigator resisted wrist flexion.^12^

### Tibialis anterior

Participant positioning: supine lying with the hip in neutral, with a sandbag/pillow on the outside of the ankle to prevent lateral hip rotation.

*Location of test site:* 33% (a third) of the distance between the tibial tuberosity and the lateral malleolus. The muscle belly (lateral to this line) was palpated during gentle resisted isometric contraction (ankle dorsiflexion).

### Myoton Recordings

The MyotonPRO device was placed on the skin perpendicular to the surface over the testing site. The device was used in multi-scan mode, which consisted of five mechanical impulses, one second apart (measurement set), giving a mean for the five measurements.^23^ Two sets of five recordings were taken at each site by the same physiotherapist. When holding the probe against the skin, a red light is present until the correct preload is reached during compression (0.18 Newtons), as well as the correct orientation of the device (perpendicular to the surface); when a green light comes on, and recording begins. The device automatically calculates the variability of the five impulses, and a coefficient of variation (CV) of 3% is used as the threshold for acceptable recordings. If the CV was >3%, the recordings were not saved and were repeated. The data were downloaded to a computer in an Excel file for later analysis.

### The Unified Parkinson Disease Rating Scale

The Unified Parkinson Disease Rating Scale (UPDRS) was also used for clinical characterisation of participants. The UPDRS is a rating tool used to monitor the progression of PD in patients.^26^ The UPDRS scale has been modified over the years by several medical organisations, including the International Parkinson's and Movement Disorders Society (MDS), producing the MDS-UPDRS.^27^ In PD clinics, the UPDRS continues to be one of the bases of treatment and research. The UPDRS scale has five segments with a series of ratings for typical Parkinson's symptoms that covers the motor and non-motor problems associated with PD.^26^ The scale is administered by a medical professional specialising in PD management. The UPDRS has a possible maximum score of 199 (due to multiple grades assigned to each extremity), indicating more disability and a least score of zero, representing no disability. On the motor segment of the UPDRS, there is a possible maximum score of 100. However, in the pre-sent study, grading multiple extremities was averaged due to unclear instructions resulting in a possible maxi-mum score of 56 instead of 100.

### Patient and Public Involvement (PPI) Activity

Three patients and four physiotherapists participated in group discussions as a PPI activity and not a research activity, which would require a qualitative methodology. The purpose was to see if the Myoton technique could be made more user-friendly to aid feasibility, and the information would be fed back to the manufacturer. A facilitator (B.E.A.S.) from the study team asked the following specific questions to aid the discussion:

**a. Patients**

Q1. How was your experience of using the device?

Q2. Is there anything you did not like about the device?

Q3. Are you interested in seeing any information that comes from the device?

Q4. If you are interested, how would you like to receive the information?

**b. Physiotherapists**

Q1. What was the most challenging aspect of collecting data?

Q2. What was the most time-consuming part of collecting data?

Q3. Were there any technical difficulties?

Q4. What type and format of information would you like to receive from the device?

### Data Analysis

#### Test of normality of data distribution

Data were assessed for normality using the Shapiro-Wilk test. Each of the two sets of data for tone, stiffness, and elasticity was examined on all three muscles, on the most and least affected sides. All data were found to be normally distributed.

#### Descriptive analysis

As the data were normally distributed, parametric statistics were applied to the data. Descriptive statistical analysis was performed using Microsoft Excel 2016 to present data in percentages, means, standard deviations and ranges. Also, descriptive characteristics of participants, such as disease dominance, were classified as tremor dominant, bradykinesia dominant and balance and stability problems dominant.

#### Reliability Analysis

The reliability of Myoton data was examined using ICC model 3,2 using SPSS (version 24). ICC values were classified as: poor below 0.50; moderate between 0.50 and 0.74; good between 0.75 and 0.89; and excellent above 0.90.^28^. The discussions with patient and health professional representatives about using the MyotonPRO device were not analysed as they did not constitute qualitative research. Their suggestions were noted, and those technical issues were communicated to the Myoton Company to improve the technology.

#### Correlation Analysis

A Pearson's correlation (r) analysis was performed between the motor scores on the UPDRS and the three Myoton parameters.

## Results

### Descriptive and demographic data

A total of 30 persons (19 males; 11 females) with PD in Hoehn and Yahr stages I - III were recruited for the study. The average disease stage was 2.35 ± 0.62 (mean ± SD) and an average age of 66.3 ± 8.9 (mean ± SD) years with a range of 47–82 years. A mean disease duration of 2.89 ± 2.18 (mean ± SD) years with a range of 4 months to nine years was recorded. Many of the participants (n = 20/30, 66.7%) presented with tremor-dominant PD. Eight out of the 30 participants (26.7%) had bradykinesia dominant, while the remaining two (6.7%) had balance and stability problems dominant PD.

### Unified Parkinson's disease Rating Scale (UPDRS)

On the motor examination section (motor score) of the UPDRS, grading of multiple extremities was averaged, resulting in a possible maximum score of 56 instead of 100 for the full test. The mean (SD) score was 19.90 (8.77), ranging from 3 (Participant PD15) up to 41 (PD11) out of 56 (Supplementary Table 1). Most participants (n=24/30, 80%) scored less than half the maximum score.

**Table 1:** Reliability results showing intraclass correlation coefficients (ICC 3,2)

Muscle	Tone (Hz)	Stiffness (N/m)	Elasticity
**Most affected side**			
**Biceps brachii**	0.94	0.95	0.92
**Flexor carpi radialis**	0.86	0.86	0.96
**Tibialis anterior**	0.93	0.96	0.97
**Least affected side**			
**Biceps brachii**	0.95	0.93	0.96
**Flexor carpi radialis**	0.79	0.85	0.73
**Tibialis anterior**	0.94	0.97	0.92

Hz = Hertz; N/m = Newton/meter

### Reliability and correlation results

All ICCs were above 0.92 for biceps brachii and tibialis anterior, demonstrating excellent reliability, and above 0.73 for flexor carpi radialis, as shown in Table 1. A Pearson's correlation analysis between the motor scores on the UPDRS and the Myoton data, recorded a moderate positive correlation with tone (r=0.62, p<0.001) and a poor positive correlation with stiffness (r=0.43, p=0.018) and a negligible negative correlation with elasticity (r=-0.13, p=0.505).^29^

### MyotonPRO Recordings

Myoton parameters recorded on the most affected side for BB showed a range of 11.3 Hz to 17.3 Hz for tone; 183.6 N/m to 305.0 N/m for stiffness and 0.90 to 2.1 for elasticity (Table 2 for summarised data and Supplementary Table 2 for individual values). However, different participants recorded the highest (PD23 and PD26) and lowest (PD3, PD6, PD28) figures for each parameter except PD23, who recorded the highest figures for tone and stiffness.

**Table 2:** Myoton parameters of the most affected side for Biceps Brachii; Flexor Carpi Radialis and Tibialis anterior muscles

Muscles	Tone Mean ± SD (Hz)	Stiffness Mean ± SD (N/m)	Elasticity Mean ± SD (log decrement)
**Biceps Brachii**	13.6±1.5	240.1±31.4	1.5 ± 0.1
	11.3–17.3*	183.6–305.0*	0.9–2.1*
**Flexor Carpi** **Radialis**	16.6±1.9	294.1±41.4	1.5 ± 0.3
	12.8–21.0*	219.2–399.0*	1.1–2.4*
**Tibialis** **Anterior**	21.3±3.5	453.9±85.0	1.5 ± 0.4
	15.9–27.6*	340.2–643.8*	0.9–2.3*

SD = Standard deviation; Ranges*

### Acceptability of Myoton technique by users

The PPI discussions indicated that the use of the MyotonPRO device was acceptable to the patient and physiotherapists. Their feedback is outlined below.

### Patients' experience

The participating patients reported that their experience using the device was satisfactory, and there was nothing they did not like about the device, except that the device did not provide an instant result that was meaningful to them. They would like to receive information about the results from the device in the form of graphical feedback. The physiotherapists taking recordings from patients reported anecdotally that patients found the device acceptable and that other patients also commented similarly to these PPI patients in the study.

### Physiotherapists' experience

The most challenging aspect of data collection was obtaining the correct orientation of the device in some cases to enable recordings to be triggered, as the device will only record when it is held perpendicular to the surface. A ‘Rotate’ message appeared on the screen when positioning was incorrect, but it did not indicate which direction (i.e. about which axis) the probe needed to be rotated to get the green light. The most time-consuming part of collecting data was to keep re-measuring if the CV was higher than 3%. Recordings were particularly difficult to obtain in one patient with oedema, as the probe kept bouncing off the hard surface of the skin.

The device had no technical difficulties, but physiotherapists expressed concern over factors that might affect the accuracy of recordings. Preferably, graphical feedback should be available directly to show the patient the results in real-time, rather than using Excel to produce graphs. The physiotherapists also suggested that a method similar to blood pressure monitoring would be very useful, giving a number for an individual in relation to normal values.

## Discussion

The aims of this study were achieved by demonstrating the feasibility of using the MyotonPRO device to measure the mechanical properties of muscle in persons with PD and establishing the within-session reliability of recordings. The MyotonPRO device was easy and quick to collect data and was considered acceptable by patients and physiotherapists.

The intraclass correlation coefficient recorded ranged from good to excellent,^28^ demonstrating the device's reliability within session in measuring muscle stiffness in the clinical setting. This finding indicates that only one set of five impulses is needed, which would reduce the time for making recordings even further. This result conforms with previous studies' findings, which reported good reliability of the MyotonPRO device among healthy^17^,^18^ and clinical populations.^12^

However, reliability between days needs to be examined to confirm that the technique can be used in the same setting to assess and monitor change over time and the effect of treatment.

Rigidity is one of the cardinal motor signs of PD, which manifests as muscle stiffness and increased tone.^1^ This symptom, like the others, also presents unilaterally and gradually progresses to affect the other side of the body, leading to poor balance, reduced physical activity and severe walking impairments.^6^ The Myoton PRO provides an objective measure of the severity of the stiffness in each limb, which could potentially inform the type, intensity and frequency of intervention appropriate for each patient with PD.

The correlation between the motor scores on the UPDRS and the Myoton data recorded a moderate correlation between UPDRS and tone (r=0.62), and a poor correlation with stiffness (r=0.48). This suggests that both the Myoton parameters would provide a crude indication of motor scores on the UPDRS but are insufficient to provide a clinically acceptable indicator in the small group of participants studied. A more comprehensive study on a larger number of participants with a broader range of severity of PD may reveal closer relationships between the clinical and Myoton measures, particularly for tone, which may prove useful as a more objective indicator of muscle health status than current subjective assessments. However, the negligible correlation between UPDRS and elasticity indicates that this Myoton parameter would not indicate motor function.

The UPDRS scores obtained for the present participants, with a mean score of 19.90 and 80% of the participants recording scores less than 28 (half of the total score), indicate that patients participating in the study had moderate disabilities, reflecting the H&Y stages (I-III). The range of UPDRS scores was illustrated by participant PD15, who recorded three as the least score, while PD11 recorded 41 as the highest score out of 56. People with PD in H&Y Stages I-III usually present with mild to moderate symptoms of the disease. The UPDRS has been shown to have ‘several ambiguities in the written text, inadequate instructions for raters, some metric flaws, and the absence of screening questions on several important non-motor aspects of PD’.^6^,^29^ Coupled with averaging of the multiple grading in the extremities, this could mask the true level of disability presented by each participant. This implies that the UPDRS may not be a very objective or reliable tool, hence the need to find an alternative measure to indicate muscle status.

The feasibility of using the MyotonPRO device in a clinical outpatient setting in Ghana was demonstrated by the fact that all 30 participants were recruited and studied within eight weeks. Discussions with patients and physiotherapists gave a useful indication of the acceptability of using the MyotonPRO device. Both user groups accepted this new technology due to the objective measure it provides and its ease of use. The manufacturer was already addressing the difficulty in finding the correct orientation in some cases to improve the device's usability. The difficulty reported in recording the patient with oedema poses an interesting challenge yet to be addressed. In addition to the practical problem of obtaining recordings, it is not yet known if the presence of fluid over the muscle gives a false reading. It is therefore important to note on the recording sheet when oedema is obvious, to help interpret the findings.

Limitations of the present study include the relatively small sample size; different H&Y stages of PD of participants; the presence of aggressive tremors; and some comorbidities, such as oedema, potentially affecting the sensitivity of the MyotonPRO device. The consultation with users was a PPI activity and did not use robust qualitative methods. A qualitative study involving more patients and therapists would be needed to help determine the acceptability and feasibility of routine clinical use of Myoton technology. Also, the UPDRS has many short-falls which may have masked the true level of disability of the participants.

The present findings warrant further studies to establish between-day reliability, obtaining data from healthy participants in Ghana for comparison with patients, and exploring potential clinical uses of the MyotonPRO, such as measuring the effects of interventions.

## Conclusion

The MyotonPRO device was reliable for measuring two sets of muscle parameters in people with PD within the same session, indicating that only one set of measurements is needed in clinical practice. Reliability needs to be established between different days of measurement. Participants with PD and physiotherapists considered the use of the MyotonPRO device to be acceptable. The successful recruitment and testing of 30 people with PD within eight weeks demonstrated the technology was feasible to use in a clinical setting in Ghana. The MyotonPRO is therefore potentially useful as an objective measure for muscle tone, stiffness and elasticity in a clinical setting. It warrants further research to establish its potential clinical utility in assessing the effect of medical and physiotherapy interventions on muscles.
